# Supplementary material for: Stakeholders’ perspectives on the status of family planning integration into differentiated antiretroviral therapy service delivery models in Uganda: A qualitative assessment
Source: PLoS One. 2025 May 22;20(5):e0324616. doi: 10.1371/journal.pone.0324616 (PMC12097553; doi:10.1371/journal.pone.0324616)
Supplement: Attachment — Submitted filename: Response_to_Reviewers.pdf [file pone.0324616.s003.pdf]

POINT-BY-POINT RESPONSE TO THE REVIEWERS' COMMENTS

PONE-D-24-53280
Stakeholders' Perspectives on the Status of Family Planning Integration into Differentiated Antiretroviral Therapy Service Delivery Models in Uganda: A Qualitative Assessment
PLOS ONE

Reviewers' Comments

Reviewer #1: This is a very comprehensive and detailed paper. It was easy to follow and the writing was clear. It was also an enjoyable paper to read as it made a strong case for integrating family planning into DSD models. In addition to capturing women's own experiences, I got a really deep understanding of the health facility-level challenges. Even though staff shortages etc are commonly reported challenges in studies such as this one, the focus on integration challenges specifically was very illuminating. Overall, the authors have done an excellent job.

Response: We thank the reviewer for this exciting compliment.

My one major concern, however, is that the paper is way too long. It would have an even greater impact and grab the reader more effectively if it was about 25% shorter. Every section is described in great detail, which can be a little too much. Consider cutting down on the methods, which are currently 6 pages long, to perhaps half the length. The discussion and/or the introduction could also be made more succinct. This is my only concern, but I will defer to the editorial policy of the journal in terms of the length of articles.

Response: We have examined all the content in the paper and dropped some statements but, unfortunately, when we added text in response to some of reviewer #2's comments, the number of pages remained largely unchanged (the total number of pages dropped by just one (1) page). We are worried that any further reductions in content will most likely distort the findings and the key messages that we are trying to put across. While we acknowledge that the paper is long; with all due respect, we would like to request that the paper length is maintained as it is at the moment.

Reviewer #2: Thank you for the opportunity to review the manuscript "Stakeholders' Perspectives on the Status of Family Planning Integration into Differentiated Antiretroviral Therapy Service Delivery Models in Uganda: A Qualitative Assessment". The authors offer a well-written account of perspectives from women living with HIV, service providers, health managers, implementing partner representatives and policymakers on FP-HIV integration from the perspective of self-care-oriented DSDM in Uganda, which gives this work a novel lens and point of view.

Response: We thank the reviewer for this important observation.

The following suggestions might help impove the manuscript:

1. Line 229. I think you meant to say you sought consent from all the clients before their participation or that participants gave their consent instead of "All clients consented before their interviews".

<u>Response</u>: We have edited the statement to read: "We sought informed consent from all the clients prior to their participation in the study". (See lines 254-255, page 9).

2. It would be helpful to have a clearer picture of the data analysis. Could you please provide more detail on the data extraction, coding, and categorisation or theme development process? Specifically, it's essential to know how many people were involved and their roles. Also, clarifying the analytical framework that guided the work would be beneficial. This would strengthen the manuscript's credibility and help address any potential concerns about bias.

<u>Response</u>: We have provided additional details to describe how the analysis of data was done, including mention of the analytical framework (Braun and Clarke, 2006). We have also included information on the people who were involved in the analysis of data at the different stages of the analysis. For details, please see page 11, page 297-311.

3. Line 329-336 – what does having FP integrated into the SC-DSDM at a given health facility mean? Did the service providers and the clients have a shared understanding of the concept?

<u>Response</u>: Ideally, at the time of the study, health facilities were expected to have integrated FP into their HIV care clinics. Clients were aware of what an integrated service was as can be seen from some of their quotations on page 11. However, to improve clarity, we have opted to write the statement about the contrast in what health providers and the clients said regarding FP integration into the SC-DSDM. Please see lines 373-375 on page 13, for details.

4. It may be an issue of preference, but I suggest presenting the results first and discussing them later. For example, the following statements could wait for the discussion section.
a. Line 389-90 – In general, evidence shows that the community-based DSD models have the lowest number of enrolled clients when compared to the other DSD models [25].

<u>Response</u>: The statement and its corresponding citation have been dropped from the revised paper.

b. Line 454 - Staff shortages have been previously reported as key barriers in the implementation of DSDM [26]

<u>Response</u>: The statement and its corresponding citation have been dropped from the revised paper.

c. Line 466-7 - Effective implementation of DSDM relies on the constant availability of ART drugs across the different ART models of care [25].

<u>Response</u>: The statement and its corresponding citation have been dropped from the revised paper

5. Line 395-96 – Is there evidence from the data to support this claim – "Our findings show that FP-HIV integration into the DSDM was not comprehensively discussed during the design of the guidelines…." Or it's the interpretation of the authors?

<u>Response</u>: Yes, there is evidence for this statement in the results section; see lines 447-449 on page 15.

6. Line 617-618 – It might help to qualify this statement and add a reference(s) – "…given the efforts put into supporting FP-HIV integration in SC-DSDM by the donor community in Uganda."

<u>Response</u>: While there was support from USAID to support FP-HIV integration, it is no longer possible to obtain a reference linked to the USAID due to President Trump's executive orders. For this reason, we have opted to revise the statement as indicated in line 709-710 on page 22.

7. Could you share the final version of the Health Facility Staff Interview Guide? The current version has changes that have not been accepted or finalised.

<u>Response</u>: Apologies for uploading a tool with track-changes. That was an oversight on our part that we fully take responsibility for. We have uploaded a clean copy of the Health Facility Staff Interview Guide, as requested.
