# Supplementary material for: Stakeholders’ perspectives on the status of family planning integration into differentiated antiretroviral therapy service delivery models in Uganda: A qualitative assessment
Source: PLoS One. 2025 May 22;20(5):e0324616. doi: 10.1371/journal.pone.0324616 (PMC12097553; doi:10.1371/journal.pone.0324616)
Supplement: S1 File — S1. In-depth interview guide for women living with HIV. S2. Interview guide for health facility staff. S3. Key informant interview guide for program managers. S4. Key informant interview guide for policymakers. (ZIP) [file pone.0324616.s001.zip › Supporting information/S1_FP-HIV Integration_Women IDI_August 20, 2022.doc]

**Women’s In-Depth Interview Guide**

**Assessment of FP-HIV Integration under Self-care Oriented Antiretroviral Therapy Differentiated Service Delivery Models in Selected Regions of Uganda**

*Objective:* 	To explore women’s perspectives and experiences in obtaining FP through DSDM

*Include:*

- Women at least 18-years-old
- Are receiving ARV care through a self-care oriented DSDM at a selected health facility
- Have ever used FP obtained from any sources since joining DSDM
- Willing to sign the informed consent
- Willing to be audio-recorded

| **BACKGROUND** | | |
| --- | --- | --- |
| **Interviewer’s name** |  | |
| **Interviewer ID number**  *Assigned during training* | [___\|___] | |
| **Interview date** | [___\|___] / [___ ___] / [___ ___]  Day Month Year | |
| **Start/End Times** | Start time [ __ __ : __ __ ] End time [ __ __ : __ __ ] | |
| **Name of region** | Central 1 region  Mid-North region  South-Western region  Central 2 region | 1  2  3  4 |
| **Name of district** | _________________________ | District #: [__\|__] |
| **Health facility** | ______________________________ | Health Facility #: [___\|___] |
| **Individual code** (choose 01 for first woman interviewed at this facility, choose 02 for second woman, etc…) | [__\|__] | |
| **Women identification number**  *Will be computed from letter representing the participant category (‘W’ for women participants), followed by the region, district, then facility and participant number* | **W –[__\|__] – [__\|__]- [__\|__] – [__\|__}**  Region District Facility Individual | |
| **Venue of interview (e.g. client’s home, health facility, etc.)** | Client’s home 1  Health facility 2  Other location (specify ________________) 3 | |
| **Language of interview** | Luo  Luganda  Runyakitara  English  other, specify _____ | 1  2  3  4  5 |

**READ:** *Today I will be asking you questions about your contraceptive use with a focus on where and how you get them. Women can get their contraceptive methods when they come to pick their ARVs at the facility or in the community. There are three different ways through which women can pick their ARVs without the need to talk to a provider. These include: a) when they go to the pharmacy to pick up their ARVs and don’t need to see the provider; b) when a health worker brings ARVs to their community for pick-up; or c) when they are in a group and only one person travels to the clinic to pick up everybody’s medicine. This study is about family planning services that are offered to clients who get their ARVs through these three different ways I described. Does that make sense?*

*I will use an audio recorder to more accurately capture what you have to say.*

**[*TURN ON RECORDER NOW*]**

*Today is [date] and I’ve just started the audio recorder for [IDI ID].  You’ve consented to participating and I’d like to get confirmation of that on the audio recording please.*

- *Do you agree to participate in this interview?*
- *Do you agree to being audio-recorded?*

**WARM UP**

1. How are you today?
2. Please tell me your age: [ ___ ___ ] years

**NOTE**: if less than 18-years, thank them for their time and end the interview

3. How many living children do you have? [ ___ ___ ]

**SECTION A: DSDM Experience**

4. How do you get your ARVs? [Probe for the three different ways through which women can receive their ARVs without the need to talk to a provider, described above].
   a. Is this the only way you have been getting your ARVs ever since you started your ART treatment?

**If yes;**

- Why did you stick with one way?

**If No;**

- Describe other ways you have been getting your ARVs.
  b. How long have you received your ARVs through the way that you are currently using to receive ARVs?
  c. What has been your experience receiving your ARVs through the way that you are currently using to receive ARVs?
  d. If the client switched ways, ask: Why did you switch back and forth between the ways?

**SECTION B: Family planning use**

5. I’d like to hear about your experience using family planning. Are you currently using any method to delay or avoid getting pregnant?

***5.1. If yes, using FP currently:***

a. What FP methods are you currently using?
b. Please tell me how you obtain the FP services and methods that you need.
   i. For **clients in the FTDR model**, ask: How and where do you obtain your FP services and methods? Probe: do you get your FP services and methods in the HIV clinic, or in the MCH/FP clinic within the facility compound but not in the HIV clinic, or are you referred for FP services and methods outside this facility?
   ii. For **clients in the CDDP model**, ask: How and where do you obtain your FP service and methods?
   iii. For **clients in the CCLAD model**, ask: How and where do you obtain your FP services and methods?
c. Do you get your FP services at the same time you get your ARVs?

***If yes, have gotten methods at the same time as getting ARVs:***

i. For **clients who receive their ARVs through facility-based models**, ask: How do you get your ARVs and FP methods at the same time? Probe: Do you get your FP methods from your ARV provider? If not, how else do you receive your FP methods at the same time as your ARVs? (e.g., are you referred to another provider but at the same facility? Escorted by provider to another provider? Sent to the pharmacy at the facility?)
ii. For **clients who receive their ARVs through community-based models**, ask: How are you able to receive your ARVs and FP methods at the same time? Do you get your FP services and methods from your ARV provider? If not, how else do you receive your FP methods at the same time you receive your ARVs?
iii. What do you like about getting FP services the same way you get your ARVs? Probe for ease of access, fewer visits to providers, counselling, time spent, ease/convenience.
iv. What do you not like about getting FP services the same way you get ARVs? Probe for counselling, long waiting time
v. What are your suggestions to improve the FP services you get the same way you get your ARVs?

***If no, have never gotten methods at the same time as getting ARVs:***

i. Are FP methods available from your ARV provider?
ii. If yes, why have you not gotten your FP method from your ARV provider?
iii. If no, would you get your FP method from your ARV provider if it was an option? Why or why not?

***5.2. If no, not currently using FP***:

a. Why are you not currently using FP?
b. What FP methods have you used in the past?
c. Please tell me how you obtained the FP services and methods that you used in the past.
   i. For **clients in the FTDR model**, ask: How and where did you obtain the last FP services and methods that you used? Probe: did you get them from the HIV clinic, or in the MCH/FP clinic within the facility compound but not in the HIV clinic, or were you referred for FP services and methods outside this facility?
   ii. For **clients in the CDDP model**, ask: How and where did you obtain the last FP services and methods that you used?
   iii. For **clients in the CCLAD model**, ask: How and where do you obtain the last FP services and methods that you used?
d. Please think back to the past year, have you gotten your FP services and methods at the same time that you got your ARVs?

***If yes, have gotten methods at the same time as getting ARVs:***

i. For **clients who receive their ARVs through facility-based models**, ask: How did you get your ARVs and FP methods at the same time? Probe: Did you get your FP methods from your ARV provider? If not, how else did you receive your FP methods at the same time as your ARVs? (e.g., were you referred to another provider but at the same facility? Escorted by provider to another provider? Sent to the pharmacy at the facility?)
ii. For clients **who receive their ARVs through community-based models**, ask: How were you able to receive your ARVs and FP methods at the same time? Did you get your FP methods from your ARV provider? If not, how else did you receive your FP methods at the same time you received your ARVs?
iii. What did you like about getting FP services the same way you get your ARVs? Probe for ease of access, fewer visits to providers, counselling, time spent, ease/convenience
iv. What did you not like about getting FP services the same way you get ARVs? Probe for stock outs, provider knowledge, long waiting time
v. What are your suggestions to improve the FP services you have gotten the same way you get your ARVs?

***If no, have not gotten methods same time as getting ARVs:***

i. Are FP methods available from your ARV provider?
ii. If yes, why have you not gotten your FP method from your ARV provider?
iii. If no, would you get your FP method from your ARV provider if it was an option? Why or why not?

**SECTION C: Wrap-up**

6. As we come to the end of this interview, what would you say has been your experience receiving your FP services and methods through the different ways that you have told me about? What has been easy for you and what have you found difficult?
7. Basing on your experience receiving ARV and FP services, what final suggestions or recommendations do you have to improve the FP services provided through ARV providers or to start providing FP services through ARV providers if services aren’t offered yet?

**Before we end our interview, do you have any final thoughts or suggestions for us?**

**Thank you for your time. We appreciate the answers you have given us.**

| **Template for Interview Notes** | |
| --- | --- |
| The overall demeanor of the participants – for example, describe if the participants were sad, uninterested, forthcoming, timid, relaxed, defensive, etc. |  |
| Any interruptions that occurred during the IDI |  |
| Cultural factors mentioned that would be important for others outside the local context to understand |  |
| Questions that were difficult for the participants to understand and suggest modifications |  |
| Issues and/or participant questions that need to be followed-up. |  |
| Any information that contradicts with what you have learned in other interviews. |  |
| Themes or patterns that are emerging [e.g. information that you are hearing over and over again from IDI participants]. |  |
| Additional general notes |  |
