# Supplementary material for: Stakeholders’ perspectives on the status of family planning integration into differentiated antiretroviral therapy service delivery models in Uganda: A qualitative assessment
Source: PLoS One. 2025 May 22;20(5):e0324616. doi: 10.1371/journal.pone.0324616 (PMC12097553; doi:10.1371/journal.pone.0324616)
Supplement: S1 File — S1. In-depth interview guide for women living with HIV. S2. Interview guide for health facility staff. S3. Key informant interview guide for program managers. S4. Key informant interview guide for policymakers. (ZIP) [file pone.0324616.s001.zip › Supporting information/S2_Interview_Guide_Health_Facility_Staff_August 20, 2022.doc]

**Health Facility Staff Interview Guide**

**Assessment of FP-HIV Integration under Self-care Oriented Antiretroviral Therapy Differentiated Service Delivery Models in Selected Regions of Uganda**

*Objective:*

- To describe FP integration into DSDM at the facility level
- To explore perspectives on barriers to and facilitators of implementing FP integration into DSDM

*Include:*

- Age 18 or older
- Works at private or public health facility (level 3 or higher)
- Has knowledge on care delivery in at least one self-care oriented DSDM available at the facility
- Willing to sign the informed consent
- Willing to be audio-recorded

| **BACKGROUND** | | |
| --- | --- | --- |
| **Interviewer’s name** |  | |
| **Interviewer ID number**  *Assigned during training* | [___\|___] | |
| **Interview date** | [___\|___] / [___ ___] / [___ ___]  Day Month Year | |
| **Start/End Times** | Start time [ __ __ : __ __ ] End time [ __ __ : __ __ ] | |
| **Name of region** | Central 1 region  Mid-North region  South-Western region  Central 2 region | 1  2  3  4 |
| **Name of district: __________________________________ District #: \|____\|____\|** | | |
| **Name of health facility**: _______________________________________ **Health Facility#:** \|____\|____\| | | |
| **Health facility level** | Special category  HC III  HC IV  General Hospital  Regional referral hospital | 1  2  3  4  5 |
| **Category of health facility** | Category 1: Provides FP services and methods (counselling, fertility awareness methods and short-term methods other than condoms) at the ART clinic  Category 2: Provides FP services and methods (counselling, fertility awareness methods and short-term methods) outside the Art clinic but in the compound within the FP/MCH clinic  Category 3: No FP services onsite | 1  2  3 |
| **Position held by respondent** | Doctor  Clinical officer  Nurse  Other | 1  2  3  4 |
| **Provider identification number**  *Will be computed from letter representing the participant category (HFS), followed by the region, district, facility and participant number* | **HFS– [__\|__]­ - [__\|__]- [__\|__]-\|__\|__\|**  Region District Facility Participant | |
| **Setting of interview**  **(specify location, e.g. office)** | ☐ Office  ☐ Other location (specify location: ________________) | |
| **Age** | [ __ __ ] | |

**READ:**

*Good morning/afternoon/evening. Thank you for speaking with me today. As I mentioned, I would like to talk to you about the provision of FP services to patients in the differentiated service delivery models for HIV (or DSDM).  I’m primarily interested in discussing three different DSDM today- Fast-track drug refills, community-based drug distribution points, and community client led ART delivery. I will also be asking about other DSDMs, where appropriate. I will use an audio recorder to more accurately capture what you have to say. Do you have any questions before I begin?*

**[*TURN ON RECORDER NOW*]**

*Today is [date] and I’ve just started the audio recorder for [IDI ID].  You’ve consented to participating and I’d like to get confirmation of that on the audio recording please.*

*Do you agree to participate in this interview?*

*Do you agree to being audio-recorded?*

**WARM UP**

1. What is your title and role at this facility?
2. Tell me about the differentiated models of ART service delivery (DSDM) that you implement at this facility. How are your patients organized across the different DSDMs? What informed/informs your decision to organize the patients in the way you described?
3. HIV patients have other non-HIV needs like all of us do. What non-HIV services do you provide to patients in HIV care at this facility? How are these services provided to patients in the different DSDMs? **Interviewer**: If not mentioned, probe: please tell me about the availability of FP services at this health facility.

**SECTION A: Provision of FP services and methods to patients in self-care oriented DSDMs**

4. Our primary discussion today will focus on the provision of FP services and methods to patients in HIV care under three self-care oriented DSDM at this facility, that is: FTDR, CDDP and CCLAD. I would like us to talk about how patients in each self-care oriented DSDM access FP services.
a) How often do you talk about family issues when giving health talks to HIV clients?
b) At what point are clients in DSDM given information about FP?
i. For **clients in the FTDR model**, ask: How and where do clients in this model obtain their FP services and methods? Probe: are FP services and methods provided in the HIV clinic, or in the MCH/FP clinic within the facility compound but not in the HIV clinic, or are clients referred for FP services and methods outside this facility?
ii. For **clients in the CDDP model**, ask: How and where do clients receiving HIV care through this model obtain their FP service and methods? What modifications in service delivery have you made to ensure that clients under this model get the FP services and methods that they need?
iii. For **clients in the CCLAD model**, ask: How and where do clients receiving HIV care via this model obtain their FP services and methods? What modifications in service delivery have you made to ensure that clients under this model get the FP services and methods that they need?
5. What FP services and methods are offered through each of the three self-care-oriented DSDM? For each DSDM, probe for:
   - counselling only,
   - short acting methods (condoms, pills, emergency contraception, injectables),
   - long-acting methods (IUD and implants),
   - permanent methods (sterilization), and
   - fertility awareness methods (Standard Days Method and any others mentioned)
6. Where do you obtain the FP commodities distributed to patients in the different self-care oriented DSDMs? For each DSDM, probe for:

- National Medical Stores
- FP-MCH clinic
- Pharmacy at the facility
- Implementing partner

7. Have the FP services offered through the different self-care oriented DSDMs changed over time? How have they changed? For each DSDM, probe for:
   - where methods are available,
   - which methods are provided (methods-mix)
   - who provides the methods and services
   - how methods are provided
   - who methods are available to,
   - other changes based on programmatic roll out
8. Where are mother-baby care points located at this facility? Probe for;
   - Art clinic
   - MCH clinic
9. How are FP services provided to mothers attending mother-baby care points?
   - ***Probe*** for various ways through which these mothers get access to FP services
     - referral within facility,
     - referral to another facility,
     - by provider at the mother-baby care point,

- by pharmacy,

10. What challenges do mothers in mother-baby care points face when accessing FP services?

**SECTION B: FP-HIV integration process**

11. Are you aware of the DSDM guidelines?
    - If yes, what do the guidelines say about the provision of FP services to patients in the different DSDMs? Probe for what the guidelines say with regard to provision of FP services in each self-care oriented DSDM.
12. The DSDM guidelines require that clients receive a package of integrated services, including FP services.
    a. What is your opinion about provision of FP services in the ART clinic?
    b. Given the way FP services and methods are provided at this facility, would you say that FP services and methods are integrated into the different DSDMs? Why or why not?
    c. What other forms of FP and HIV integration exist at this facility?
    d. What challenges do DSDM clients face when accessing FP services and methods at other points other than through the DSDMs? How can these challenges be minimized?
13. What factors enable you to implement FP integration into DSDM in accordance with the guidelines? What barriers do you face?
    - What adaptations to the guidelines do you make to provide FP services to DSDM clients and why?
    - To what extent have HIV care health providers been trained to offer integrated FP-HIV services? Who conducted the training? How often is the training done?
    - How do HIV care health providers feel when they have to provide FP services and methods in the different DSDMs when the same services are available in the MCH/FP clinic? How does this affect FP service delivery?
14. What have been the key challenges of integrating FP into DSDMs?
    - Are there challenges that face all the DSDM? What are they?
    - Are there any challenges specifically related to training health providers on provision of integrated FP-HIV services?
    - Are there challenges that are specific to each DSDM?
    - How has FP-HIV integration affected the clients?
    - What was done to address these challenges?
15. What are some of the strategies that have been put in place that facilitate and help to sustain FP-HIV integration at your facility?

**Probe for:**

- Changes of the work environment
- Changes of consultation schedules
- Staffing
- Training
- Supervision
- Documentation/monitoring
- Resource mobilization Commodity security.

16. How has the integration of FP into DSDM impacted your day-to-day work?
    - What do you like and dislike about providing integrated services?
17. Who supports this health facility to integrate FP services into DSDMs? Probe for:
    - Ministry of Health
    - Implementing partner
    - Both ministry of health and implementing partner
    - Explain the nature of support you receive
    - Monetary support
    - Staff secondment
    - Infrastructure
18. Is the support provided the same across all DSDMs for stable patients? Are there differences in the support for community-based DSDMs and facility-based DSDMs?

**SECTION C:** **Recommendations**

19. What lessons have you learned so far with the way FP services have been integrated into HIV care services under the different DSDM? Based on these lessons, what aspects of the integration would you propose to keep (moving forward)? What aspects would you recommend to modify/change? Are there ways integration could be more streamlined?
20. From your experience providing integrated FP-HIV services, would you recommend the government or implementing partners to scale up FP-HIV DSDM integrated services? Why or why not?

**Before we end our interview, do you have any final thoughts or suggestions for us?**

**Thank you for your time. We appreciate the answers you have given us**

| **Interview Notes** | |
| --- | --- |
| The overall demeanor of the participants – for example, describe if the participants were sad, uninterested, forthcoming, timid, relaxed, defensive, etc. |  |
| Any interruptions that occurred during the IDI. |  |
| Cultural factors mentioned that would be important for others outside the local context to understand. |  |
| Questions that were difficult for the participants to understand and suggest modifications. |  |
| Issues and/or participant questions that need to be followed-up. |  |
| Any information that contradicts with what you have learned in other interviews. |  |
| Themes or patterns that are emerging [e.g. information that you are hearing over and over again from IDI participants]. |  |
| Additional general notes |  |
