# Supplementary material for: Stakeholders’ perspectives on the status of family planning integration into differentiated antiretroviral therapy service delivery models in Uganda: A qualitative assessment
Source: PLoS One. 2025 May 22;20(5):e0324616. doi: 10.1371/journal.pone.0324616 (PMC12097553; doi:10.1371/journal.pone.0324616)
Supplement: S1 File — S1. In-depth interview guide for women living with HIV. S2. Interview guide for health facility staff. S3. Key informant interview guide for program managers. S4. Key informant interview guide for policymakers. (ZIP) [file pone.0324616.s001.zip › Supporting information/S3_Key_Informant_Interview_Guide_Program_Managers_Aug 20, 2022.doc]

**Key Informant Interview Guide for Program Managers**

**Assessment of FP-HIV Integration under Self-care Oriented Antiretroviral Therapy Differentiated Service Delivery Models in Selected Regions of Uganda**

*Objectives:*

- To document the various approaches to FP integration into DSDM
- To describe the process of implementing and monitoring the national guidelines regarding FP integration into DSDM

*Include:*

- Age 18 or older
- District Health Officers, District FP focal persons, or senior technical staff at implementing partner organizations who have knowledge of FP integration into self-care oriented DSDM
- Experience implementing or supporting FP integration into self-care oriented DSDM
- Serves in a district within selected regions
- Willing to sign the informed consent
- Willing to be audio-recorded

| **BACKGROUND** | | |
| --- | --- | --- |
| **Interviewer’s name** |  | |
| **Interviewer ID number**  *Assigned during training* | [___\|___] | |
| **Interview date** | [___\|___] / [___\|___] / [___\|___]  Day Month Year | |
| **Start/End Times** | Start time [ __ __ : __ __ ] End time [ __ __ : __ __ ] | |
| **Name of region** | Central 1 region  Mid-North region  South-Western region  Central 2 region | 1  2  3  4 |
| **Name of district: _____________________________________ District #: \|___\|___\|** | | |
| **Interview number**  *Will be assigned sequentially* | \|___\|___\| | |
| **Program Manager identification number***  *Will be computed from the letter representing the participant category (PM) followed by participant number* | **PM – [__\|__]__\|__\|**  **Dist#** Participant # | |
| Category of participant | District official (DHO/ADHO-MCH/FP Focal person) .......1  Technical Officer working a regional IP ...........................2 | |
| **Setting of interview (Specify location, e.g. office)** | ☐ Office  ☐ Other location (specify:______________) | |

**READ:**

*Good morning/afternoon/evening. Thank you for speaking with me today. As I mentioned, I’d like to talk about differentiated service delivery models for ARV refills, or DSDM. As part of this study, we are conducting interviews with program managers to understand how the integration of FP into the DSDM is happening. Does this explanation make sense? Do you have any questions before I begin?*

*I will use an audio recorder to capture more accurately what you have to say.*

**[*TURN ON RECORDER NOW*]**

Today is [date] and I’ve just started the audio recorder for [KII ID].  You’ve consented to participating and I’d like to get confirmation of that on the audio recording please.

- Do you agree to participate in this interview?
- Do you agree to being audio-recorded?

**WARM UP QUESTIONS**

1. What role do you play in the District Health Office/Partner organization?
   - How long have you served in this role?
2. Tell me about the status of family planning service delivery in this district. Which population groups do you target? Which population groups do you usually miss?

**SECTION A: IMPLEMENTATION OF DSDM GUIDELINES**

3. The *Implementation Guide for Differentiated Service Delivery Models of HIV and TB Services in Uganda* states that family planning should be offered to DSDM clients along with other non-HIV services. Can you describe the process used to implement the guidelines especially as they pertain to the provision of FP services and methods to clients in the different DSDMs?
   - How was/is the guideline implemented at each level of the health system?
   - Who was/is involved? Trained?
   - What is your opinion regarding the provision FP services to DSDM clients along with other services?

**SECTION B: DSDM GUIDELINES IMPLEMENTATION**

4. Could you please describe how integration of FP into DSDM is meant to take place in the health facilities?

**Probe for:** both facility-based and community-based models

- Which types of DSDM were supposed to include FP services?
- What types of clients were supposed to be offered FP through DSDMs
- When are clients supposed to be offered FP through DSDM?
- What FP services were supposed to be available through DSDM?
- How were clients intended to be made aware of the integration?
- How were monitoring activities intended to be conducted?
- What health-systems adjustments were intended to be made to support integration—e.g. any changes to the supply chain (the procurement, transportation, and storage of commodities), or how providers would counsel clients, or how service provision would be tracked, or how data would be collected (using new forms) or entered?

5. To what extent was the plan for integrating FP into DSDMs followed?

- Please explain the range of approaches to FP integration into DSDM that occur in practice
- What guidelines were followed during the process of integrating FP into DSDMs?
- Was the FP-HIV integration plan implemented as per the original plan?
- If not, what are the reasons for not adhering to the intended plan? **Probe for** training, staff mobility, supervision, territorial conflict across programs, overwork
  - What adaptations were made to the original FP-HIV integration plan, and what informed them? Any unforeseen systems-level changes or adaptations that had to be made?

6. Can you describe how the implementation of the *Implementation Guide for Differentiated Service Delivery Models of HIV and TB Services in Uganda* is being monitored?
   a) ***To the DHO/Assistant DHO-MCH:***

- How often are health facilities supported to implement the DSDM guidelines s expected? Who supports them? How are they supported?
- How do clients in the different DSDMs access the FP services and methods that they need?
- What reporting is required at the facility level? District level? National level?
- Where and what do health facilities report, and how often do they report?
- Are any FP-specific indicators collected? What indicators? Where are they reported?
  b) ***To the FP Focal Person***
- What FP services and methods do you provide to HIV-patients in the different self-care oriented DSDMs – i.e. HIV patients in FTDR, CDDP and CCLAD? Is there any special arrangement for HIV-positive patients who receive their ART drugs in the HIV clinic?
- How do patients in the community-based DSDMs (CDDP and CCLAD) access their FP services and methods?
- How do you ensure that clients in the different DSDMs have full and unlimited access to the FP services and methods that they need?
  c) ***To the technical officer from an implementing partner (IP) organization***
- Please tell me about the support that you (the IP) provide to the health facilities under your jurisdiction to assist them in implementing the DSDM guidelines as expected.
- To what extent do facilities integrate FP services and methods within the different DSDMs?
- What forms of FP integration do you support?

7. Now let’s discuss what helped the process of implementing FP integration into DSDMs according to the guidelines– what are you aware of that made the process easier and helped it along?
   - Are facilitators different for different models of DSDM? Facility vs. community models?
8. Now let’s discuss the challenges and barriers that were faced during the process of implementing FP integration into DSDMs according to the guidelines– what challenges or barriers are you aware of?
   - Are challenges and barriers different for different models of DSDM? Facility vs. community models?
   - What was done to address these challenges?
   - How well has this worked?

**SECTION C:** **Recommendations**

9. From your experience in supporting the integration of FP services in DSDM, what works well for FP-HIV integration? What are the problems or challenges that still need to be resolved? (If problems or challenges are mentioned, probe for possible solutions.)
10. From your experience piloting/implementing/evaluating FP-HIV integrated services, under what circumstances would you recommend the government to scale up FP-HIV integrated services in DSDMs?

**Before we end our interview, do you have any final thoughts or suggestions for us?**

**Thank you for your time. We appreciate the answers you have given us**

| **Template for Interview Notes** | |
| --- | --- |
| The overall demeanor of the participants – for example, describe if the participants were sad, uninterested, forthcoming, timid, relaxed, defensive, etc. |  |
| Any interruptions that occurred during the IDI |  |
| Cultural factors mentioned that would be important for others outside the local context to understand |  |
| Questions that were difficult for the participants to understand and suggest modifications |  |
| Issues and/or participant questions that need to be followed-up. |  |
| Any information that contradicts with what you have learned in other interviews. |  |
| Themes or patterns that are emerging [e.g. information that you are hearing over and over again from IDI participants]. |  |
| Additional general notes |  |
