# Supplementary material for: Stakeholders’ perspectives on the status of family planning integration into differentiated antiretroviral therapy service delivery models in Uganda: A qualitative assessment
Source: PLoS One. 2025 May 22;20(5):e0324616. doi: 10.1371/journal.pone.0324616 (PMC12097553; doi:10.1371/journal.pone.0324616)
Supplement: S1 File — S1. In-depth interview guide for women living with HIV. S2. Interview guide for health facility staff. S3. Key informant interview guide for program managers. S4. Key informant interview guide for policymakers. (ZIP) [file pone.0324616.s001.zip › Supporting information/S4_Key_Informant_Interview_Guide_Policymakers_Aug 20, 2022.doc]

**Key Informant Interview Guide for Policymakers**

**Assessment of FP-HIV Integration under Self-care Oriented Antiretroviral Therapy Differentiated Service Delivery Models in Selected Regions of Uganda**

*Objectives:*

- To describe the process of developing, implementing, and monitoring the national guidelines regarding FP integration into DSDM

*Include:*

- Age 18 or older
- National or district level health officials with expertise in FP integration into HIV care
- Experience in policy design, implementation, or monitoring
- Willing to sign the informed consent
- Willing to be audio-recorded

| **BACKGROUND** | | |
| --- | --- | --- |
| **Interviewer’s name** |  | |
| **Interviewer ID number**  *Assigned during training* | [___\|___] | |
| **Interview date** | [___\|___] / [___\|___] / [___\|___]  Day Month Year | |
| **Start/End Times** | Start time [ __ __ : __ __ ] End time [ __ __ : __ __ ] | |
| **Interview number**  *Will be assigned sequentially* |  | 1  2  3 |
| **Policy maker identification number***  *Will be computed from the letter representing the participant category (POL) followed by the participant number* | **POL– [__\|__]**  Participant # | |
| **Setting of interview (Specify location, e.g. office)** | ☐ Office  ☐ Other location (specify:________________________) | |

**READ:**

*Good morning/afternoon/evening. Thank you for speaking with me today. As I mentioned, I’d like to talk about differentiated service delivery models for ARV refills, or DSDM. As part of this study, we are conducting interviews with policymakers to understand how the integration of FP into the DSDM was conceptualized, and the process of developing, implementing and monitoring the integration process. This information will help to inform our understanding of how the integration of FP into HIV care was meant to be implemented and monitored. Does this explanation make sense? Do you have any questions before I begin?*

*I will use an audio recorder to capture more accurately what you have to say.*

**[*TURN ON RECORDER NOW*]**

Today is [date] and I’ve just started the audio recorder for [KII ID].  You’ve consented to participating and I’d like to get confirmation of that on the audio recording please.

- Do you agree to participate in this interview?
- Do you agree to being audio-recorded?

**WARM UP QUESTIONS**

1. What role do you play in the MOH?
   - How long have you served in this role?
2. What is the current status of DSDM implementation in Uganda?
   - Do you have any involvement in family planning integration into DSDMs?
   - How long have you been involved in this way?

**SECTION A: PROCESS FOR DEVELOPING THE DSDM GUIDELINES AND PROVISION OF NON-HIV SERVICES TO DSDM CLIENTS**

3. I would like to learn more about the *Implementation Guide for Differentiated Service Delivery Models of HIV and TB Services in Uganda*. What was the process used to develop these guidelines?
   - What necessitated the development of guidelines on DSDM?
   - Who was involved in the development of these guidelines?
   - What was your role in the development of these guidelines?
4. The *Implementation Guide for Differentiated Service Delivery Models of HIV and TB Services in Uganda* states that family planning should be offered to DSDM clients along with other non-HIV services. How did the policymakers envisage FP services being provided to clients in the different DSDMs?
   - What types of FP services did the policymakers envisage being provided to the clients in the different DSDMs? **Interviewer**: Please focus on **self-care oriented** DSDMs.
   - Who was involved in decisions regarding the types of FP services to offer in each DSDM?
   - What factors did policymakers consider in deciding on the types of FP services offered in each DSDM? Were these factors different for each DSDM or types of DSDM (i.e., facility vs. community-based)?

**SECTION B: DSDM GUIDELINES IMPLEMENTATION**

5. Can you describe the process used in rolling out the *Implementation Guide for Differentiated Service Delivery Models of HIV and TB Services in Uganda* to the different health facilities in the country?
   - How was the roll-out of the guidelines implemented at each level of the health system?
   - Who was involved? Who was trained? Who led the trainings?
   - To what extent did the roll-out plan include emphasis on the need to integrate FP into the different DSDMs? Interviewer: Probe for each DSDM.
   - What types of FP integration did the roll-out plan emphasize to the health providers? Specifically, what were the providers told to do when it comes to FP integration into the different DSDMs?
6. Could you please describe how integration of FP into DSDM is meant to take place in the health facilities?

**Probe for:** both facility-based and community-based models

- What FP services and methods were supposed to be available through DSDM? All methods? If only some, which ones?
- How were monitoring activities intended to be conducted?
- What health-systems adjustments were intended to be made to support integration—e.g. any changes to the supply chain (the procurement, transportation, and storage of commodities), or how providers would counsel clients, or how service provision would be tracked, or how data would be collected (using new forms) or entered?

7. To what extent was the plan for integrating FP into DSDMs followed?

- Please explain the range of approaches to FP integration into DSDM that occur in practice
- What guidelines were followed during the process of integrating FP into DSDMs?
- How did/does the actual integration differ from the original plan? What informed the adaptations in the original plan?
- What are the reasons for not adhering to the intended plan?
  - **Probe for** training, staff mobility, supervision, territorial conflict across programs (e.g. FP services being a main preserve of the MCH/FP clinic but not the HIV clinic), work overload
- Any unforeseen systems-level changes or adaptations that had to be made?

8. Can you describe how the implementation of the *Implementation Guide for Differentiated Service Delivery Models of HIV and TB Services in Uganda* is being monitored?
   - What reporting is required at the facility level? District level? National level?
   - Where and what do the health facilities report, and how often do they report?
   - Are any FP-specific indicators collected? What indicators? Where are they collected? To whom are they reported?
9. Current status
   - What areas of FP-HIV integration are going well?
   - What areas of FP-HIV integration are lagging behind?

**SECTION C:** **WRAP UP**

10. Do you have any final thoughts you would like to share about integrating FP services into DSDM?

**Before we end our interview, do you have any final thoughts or suggestions for us?**

**Thank you for your time. We appreciate the answers you have given us**

| **Template for Interview Notes** | |
| --- | --- |
| The overall demeanor of the participants – for example, describe if the participants were sad, uninterested, forthcoming, timid, relaxed, defensive, etc. |  |
| Any interruptions that occurred during the IDI |  |
| Cultural factors mentioned that would be important for others outside the local context to understand |  |
| Questions that were difficult for the participants to understand and suggest modifications |  |
| Issues and/or participant questions that need to be followed-up. |  |
| Any information that contradicts with what you have learned in other interviews. |  |
| Themes or patterns that are emerging [e.g. information that you are hearing over and over again from IDI participants]. |  |
| Additional general notes |  |
